# Advancing the application of systems thinking in health: understanding the growing complexity governing immunization services in Kerala, India

**DOI:** 10.1186/1478-4505-12-47

**Published:** 2014-08-26

**Authors:** Joe Varghese, V Raman Kutty, Ligia Paina, Taghreed Adam

**Affiliations:** Centre for Chronic Disease Control and Governance Hub, Public Health Foundation of India, Delhi NCR, Plot No. 47, Sector 44, Gurgaon, 122002 India; Achutha Menon Centre for Health Science Studies, Sree Chitra Tirunal Institute for Medical Science and Technology, Thiruvananthapuram, India; Department of International Health, Johns Hopkins University School of Public Health, Baltimore, USA; Alliance for Health Policy and Systems Research, World Health Organization, Geneva, Switzerland

**Keywords:** Causal loop diagram, Complex adaptive systems, Governance, Immunization, India, Kerala, Systems thinking, Trust, Vensim

## Abstract

**Background:**

Governing immunization services in a way that achieves and maintains desired population coverage levels is complex as it involves interactions of multiple actors and contexts. In one of the Indian states, Kerala, after routine immunization had reached high coverage in the late 1990s, it started to decline in some of the districts. This paper describes an application of complex adaptive systems theory and methods to understand and explain the phenomena underlying unexpected changes in vaccination coverage.

**Methods:**

We used qualitative methods to explore the factors underlying changes in vaccination coverage in two districts in Kerala, one with high and one with low coverage. Content analysis was guided by features inherent to complex adaptive systems such as phase transitions, feedback, path dependence, and self-organization. Causal loop diagrams were developed to depict the interactions among actors and critical events that influenced the changes in vaccination coverage.

**Results:**

We identified various complex adaptive system phenomena that influenced the change in vaccination coverage levels in the two districts. Phase transition describes how initial acceptability to vaccination is replaced by a resistance in northern Kerala, which involved new actors; actors attempting to regain acceptability and others who countered it created several feedback loops. We also describe how the authorities have responded to declining immunization coverage and its impact on vaccine acceptability in the context of certain highly connected actors playing disproportionate influence over household vaccination decisions.

Theoretical exposition of our findings reveals the important role of trust in health workers and institutions that shape the interactions of actors leading to complex adaptive system phenomena.

**Conclusions:**

As illustrated in this study, a complex adaptive system lens helps to uncover the ‘real’ drivers for change. This approach assists researchers and decision makers to systematically explore the driving forces and factors in each setting and develop appropriate and timely strategies to address them. The study calls for greater consideration of dynamics of vaccine acceptability while formulating immunization policies and program strategies. The analytical approaches adopted in this study are not only applicable to immunization or Kerala but to all complex interventions, health systems problems, and contexts.

## Background

Organizing immunization services to protect the society against preventable diseases is a core function of public health. In India, the Universal Immunization Programme (UIP) introduced in 1985, targets 27 million infants and 30 million pregnant women every year and is one of the largest in the world [1]. Though UIP has improved the availability of vaccines and cold chain management compared to earlier immunization programs, the system has not yet achieved sustained improvement in vaccination coverage in many Indian states [2, 3]. It has been slated as a mechanistic approach, which was simplistically expected to improve immunization coverage through the improvements in health infrastructure, financing, supplies, and better management practices [4, 5]. This approach has typically failed to account for the unique characteristics, interactions, and needs within local systems and the diversity of actors impacting a household’s decision to vaccinate. Such an approach was often constrained by a lack of understanding of the complex behaviour of local health systems, which often do not respond as expected to external interventions and policies. Furthermore, such an approach can only provide a limited explanation for fluctuations in immunization coverage rates, over time.

This paper describes an application of systems thinking to understand the complex phenomena underlying changes in vaccination coverage in India. Specifically, this study seeks to use a complex adaptive system (CAS) lens to understand the features of a complex system that governs childhood immunization in parts of the Indian state of Kerala, where immunization coverage drastically declined after a period of high coverage.

**Figure 1:**
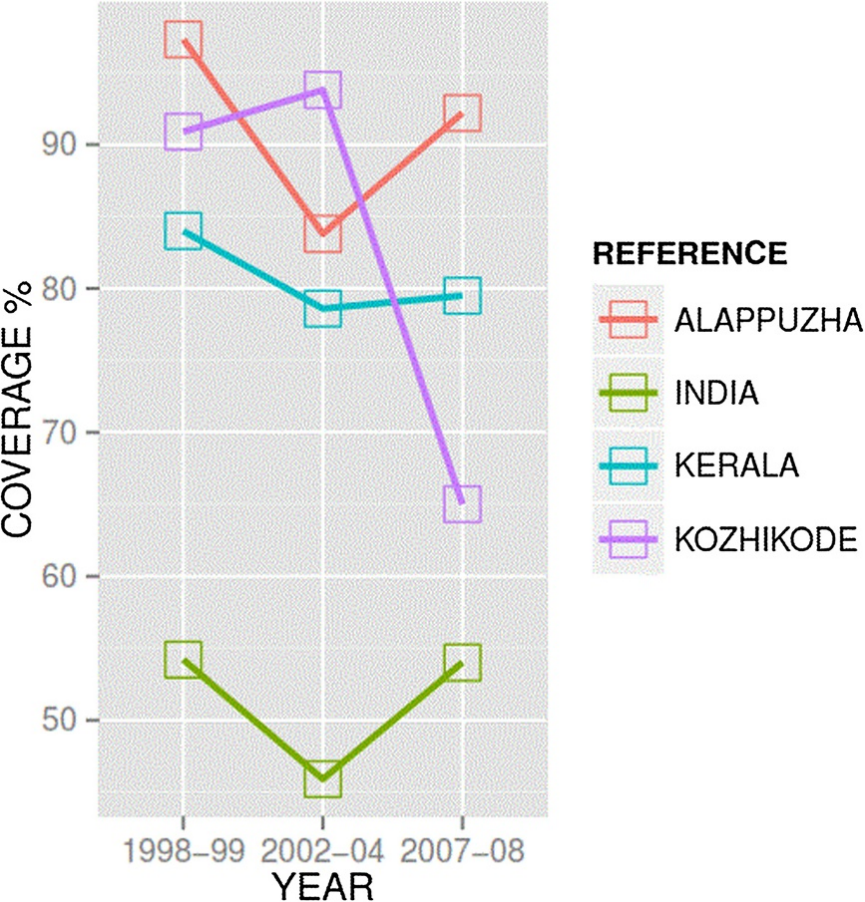
**Changes in vaccine coverage in Alappuzha and Kozhikode districts of Kerala.**

Kerala holds a special place in the global public health discourse for its remarkable health achievements despite low economic status [6]. Unlike the national average of full immunization coverage (of BCG, Polio, DPT and measles) of 54.2%, Kerala had achieved over 84% in the late 1990s before starting to decline in subsequent years (Figure 1) [7]. This decline mostly involved the northern districts of Kerala. The reduction of coverage in northern districts in Kerala is a concern for public health authorities as it negates herd immunity that protected communities against the potential spread of vaccine-preventable diseases [8, 9]. The sudden decline in immunization coverage, in a state where vaccines were uncritically accepted as a social good in the past, has puzzled public health officials and experts [10].

### Theoretical underpinnings

Our study adopted a CAS-lens for exploring the factors that influenced immunisation services in Kerala and identified many characteristics of the CAS phenomena in the discussion. CAS exhibit the capacity to self-organize, adapt, and learn from past experiences, which often results in counterintuitive and unintended effects or paradoxical behaviour [11, 12]. CAS may exhibit various unique features, such as path dependence, feedback loops, scale-free networks, emergent behaviour, and phase transition.

Path dependence refers to non-reversible processes that have similar starting points yet lead to different outcomes due to different choices made along the way. Feedback loops that feed into the system explain how small changes can grow into large consequences that can be ‘reinforcing’ a particular outcome or may lead the system to go back to an original state, what is called ‘balancing’ or goal-seeking loops. Phase transitions occur at tipping points when systems show sudden changes even without any additional inputs. Scale-free networks refer to the formation of influential hubs that can shift the focus and power of networks by exerting higher influence on other actors in the network through their multiple interconnectedness, hence activating a change in behaviour. CAS can also exhibit emergent behaviour when smaller entities jointly create a spontaneous order and show organised behaviour.

In the context of the governance of public health interventions, such as immunizations, complexity is generated from the diverse and dynamic nature of interactions between the system’s actors and the multiple interacting factors such as values, culture, history, norms, and distribution of power and information in societies. Furthermore, the interconnectedness between the various actors of the system means that their actions inevitably create ripple effects that cause continuous change and adaptation of the intervention in the entire system [13].

Exploring these complex system features in Kerala will shed light on the factors that drove the unexpected changes in immunization coverage and will provide insights into the types of system adaptations that must be considered by national immunization programs.

## Methods

A qualitative case study design was used to obtain an understanding of immunization coverage in Kerala. The data used in this paper originated from a larger study seeking to understand the governance of immunization in two states in India [14]. Initial findings from this earlier study highlighted the need to further explore the complexity of immunization services in one of the states – Kerala – where we observed surprising trends in immunization coverage (Figure 1).

Two districts in Kerala were randomly identified from high and low coverage districts in the state [7]. The districts were Alappuzha, a better performing (90.2%) district in terms of immunisation coverage as per the third District Level Health Service survey, and Kozhikode, a poor performing (65%) district. In each of these districts, better performing and poor performing areas in terms of immunisation service were identified with the help of district level managers. Though the difference in immunization coverage between two areas within the districts was marginal, the identification of different locations helped in collecting information from diverse contexts. From each area, two primary health centres and a private health facility were selected for observation of the immunisation services and interviewing the practitioners.

The main data sources included a literature and document review (including news reports), in-depth interviews, focus group discussions, and observations of immunization services. All data collection was undertaken by the first author in Malayalam (local language), over a period of six months during late 2009 and early 2010.

In each district, in-depth interviews were carried out with immunization service providers from public and private sector, those who facilitate vaccination, such as community health workers, and those who opposed it. We used a snowball sampling method, whereby, at the end of the interview, the respondent’s suggestion was asked about other important stakeholders in order to identify the next respondent. The experts interviewed were also identified using a snowball method based on their research experience on immunization or their expertise of immunization service, either as a past or present state or district level immunization program implementer.

Focus group discussions were undertaken with mothers of children below five years of age and one with the health workers of one of the primary health centres. The mothers were identified and invited with the help of community workers and the discussions were arranged in one of the local houses or local Anganwadies (pre-school and nutrition centres for women and children). The number of participants per focus group discussion varied from 7 to 10.

Participant and non-participant observations were made with the help of an observation guide in order to gather insights into cultural meanings and interpretations related to provider and beneficiary behaviours and context. All participant observations were made during the house visits that the first author made along with community health workers, aiming to mobilizing beneficiaries for vaccination. During each of the visits the researcher was introduced to households as a public health researcher and was involved in motivating and educating the families on childhood vaccinations. In most of the households visited, the initial communication related to vaccination was provided by the community health worker and the researcher was asked to clarify when further explanation was required. For participant observation, the researcher had to play the role of a public health expert and researcher simultaneously. This involved active engagement in mobilizing the parents for immunization of their children along with making qualitative observations from this engagement for the research. Non-participant observations were made during immunisation sessions at health facilities, outreach immunisation sessions, and review meetings of field staff in charge of the immunisation programme. Important observations were noted onsite and, at the end of the day, a full record of the field notes was prepared by appropriately commenting on each of these activities.

All interviews and focus group discussions were digitally recorded, transcribed, and translated into English. Content analysis was applied to the transcripts of interviews and focus group discussions, as well as the field notes of observations [15]. The various categories for content analysis, as informed by the application of a CAS lens, were identified prior to the analysis. Using these categories, we used a deductive coding of the data. Atlas.ti ver.7 was used for arranging the text according to codes and managing the codes in the interpretive phase.

The three different methods were used for data collection from various types of respondents; observation, interviews, and focus groups involving various sources of information helped in triangulating the findings. In order to reduce the subjective bias of the first and second authors due to their prior information of the functioning of Kerala’s health system, a peer scrutiny of the analysis was done by the third author who assessed the assumptions made.

Based on the qualitative data analysis, a causal loop diagram (CLD) was developed using Vensim PLE [13, 16]. CLDs are qualitative representations of underlying mental models and are typically used to illustrate feedback and interactions among health system actors [17]. For this study, the purpose of the CLD was to assist in the identification and interpretation of the feedback loops that emerged in the context of immunization. The CLD was also used to guide the brainstorming discussion among authors about other complex phenomena that governed the analysis period. The variables used in the CLD were derived from the qualitative data, as well as from the literature on determinants of immunization coverage. The CLD uses standard notation, where positive arrows denote that two variables change in the same direction, and negative arrows denote that two variables change in opposite directions. An arrow with a double hash mark on it (||) indicates that there is a time delay in the relationship denoted. Reinforcing loops, which indicate that variables have an overall amplifying effect, are labelled with an “R” and a loop symbol. Balancing loops, which indicate that variables have an overall dampening effect, are labelled with a “B” and a loop symbol. The loop symbol is either clockwise or counter-clockwise, depending on the direction in which the loop is read [17]. Where there were multiple loops, we numbered them in the order in which they appear in the text. We used the CLDs not only as a summary of the content analysis, but also to conceptualize and develop additional potential linkages between factors. Dotted arrows have been used to denote those potential additional relationships that were not empirically explored.

The study protocol was reviewed for ethical and technical clearance by the Institutional Review Board (Sree Chitra Tirunal Institute for Medical Science and Technology, Thiruvananthapuram, India). Written permission for data collection was obtained from state level health officials as well as from district level officials and participation in the study was made voluntary by ensuring informed consent from all participants and the possibility to withdraw at any time. All identifiers of the study participants from the transcripts of the data were removed by the first author to ensure anonymity of the study participants.

## Results

The fieldwork included 7 participant and 7 non-participant multi-site observations, 5 focus group discussions, and 17 interviews with beneficiaries, community intermediaries (community health workers, nutrition and pre-school teachers and community leaders), and providers from public and private sector. The study also involved key informant interviews with 6 experts.

As described in the introductory section, our analysis of trends in immunization coverage in both districts showed a sudden decline in immunization coverage in Kozhikode; based on three rounds of the District Level Household and Facility Survey, Kozhikode showed a decline after the second round of the survey in the 2002–2004 period. The full immunization coverage in Kozhikode district in northern Kerala dropped from 94% (2002–2004) to 65% (2007–2008). During the same period, the coverage in a southern district, Alappuzha, had in fact gone up from about 84% to around 92%. The decline in immunization coverage in Kozhikode is, in fact, a reversal of the trend from the earlier period between the first (1998–1999) and the second survey (2002–2004), which showed improvement in vaccination coverage.

The qualitative data showed a widespread hesitancy against routine vaccinations in Kozhikode district, while routine vaccinations are widely accepted in Alappuzha district. It was also observed that the resistance against vaccination was often limited to geographical locations. It was observed during the house visits in vaccine-resistant areas of Kozhikode district that most of the unvaccinated children are found in households of close geographical vicinities. The differences in immunization coverage in different areas within Kozhikode district is explained by the spread and extent of vaccine-resistant geographical locations within the district. However, we could not elicit major differences in vaccine acceptability between high and low coverage areas of Alappuzha district, which may be explained by other factors such as poor socioeconomic status of the region, absence of public health human resources, or anomalies in the reporting of vaccination coverage.

In the following section, we first illustrate the feedback loops that emerged as a result of interactions among the key actors and contributed to phase transitions from vaccination acceptance to resistance. We introduce two separate CLDs that are relevant to the acceptability phase and vaccine-resistance phase to discuss the contrasting features of these two phases. We also describe the feedback that affected the districts differently after showing a high level of vaccine acceptability in the beginning. Next, we show how the authorities have responded to this problem of decline in immunization coverage and discuss the impact of their response in the presence of certain highly connected actors playing a disproportionate influence over a household’s vaccination decision.

### Phase 1: Acceptability

**Figure 2:**
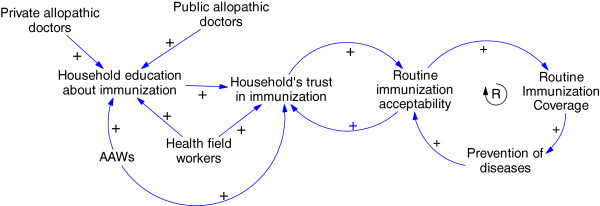
**Causal loop diagram representing the phase of high vaccine acceptance and coverage.**

As mentioned in the introduction, the UIP heralded a shift compared to earlier programs in both the availability and acceptability of immunizations in Kerala. After a decade of implementation, society perceived vaccines to be effective in the prevention of certain diseases and coverage increased significantly. Figure 2 displays the CLD illustrating the factors promoting the acceptability of immunization under UIP. There were several actors who contributed to this. Public allopathic doctors were important sources of health education and encouraged immunization. Private sector allopathic doctors also contributed to this effort either in collaboration with UIP or through their independent efforts. A large part of the success of the UIP program during this period was credited to the joint efforts of health field workers and anganwadi workers (AWW). These two groups belonged to different sectors – field-staff are deployed by the public health department and the AWW, the pre-school educator and nutrition worker belonged to the Integrated Child Development Program. Field-staff fostered acceptability to vaccination through their regular house visits and constant interactions with mothers, and AWWs increased the community’s awareness of immunisation programmes. The vaccine literacy of the households was increased not only by the constant interactions of these two workers with the households, but also because of their status in the community as a trusted source of health information.

Within a household, the mothers played a significant role in vaccination decisions as she held most of the vaccination-related information. The media contributed by carrying positive messages about immunization to community households. In the CLD depicting the reinforcing loop displayed in Figure 2 we also observed that the prevention of diseases through vaccines reinforced the household’s acceptability to immunization. The widespread acceptability of vaccines is reflected by the absence of major opposition to immunization programs during this phase, as well as by a significant increase in the overall vaccination coverage (Kerala = 84%; national average = 54.2%) [18].

In a push for polio eradication, a polio vaccination campaign was introduced in 1995 as part of the Global Polio Eradication Initiative [19] by administering additional oral polio vaccine to all children under five on two national immunization days. Despite initial opposition from some medical professionals, the campaign was generally well received, benefiting from ongoing civil society participation. The opposition was from some physicians in academic settings as well as some private doctors who questioned the need for additional oral polio vaccine for a state like Kerala, when the state already had high routine immunization coverage. Initial opposition was neutralized by the public’s confidence in vaccination in general and considerable state support for the program. For example, according to our respondents, there was extensive participation in the program implementation by several actors, including several government departments, in addition to health, as well as Panchayats (village level elected governance institution), NGOs, and schools. In 2000, a case of polio was reported in Kerala. Although this event was seen as a failure of the public system, the impact on the immunization program was not immediately evident and the efficacy or safety of the vaccine was not questioned.

### Phase 2: Opposition

**Figure 3:**
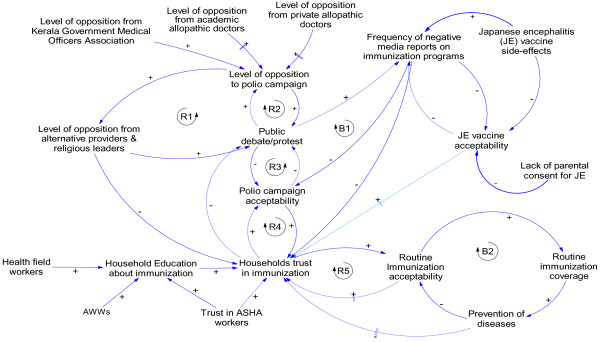
**Causal loop diagram representing the phase of low vaccine acceptance in Kerala.**

The polio case in Kerala was followed by a series of critical events related to immunization-created feedback loops that influenced sudden changes to the social acceptability of vaccines. Figure 3 displays the CLD that shows increased complexity and the new feedback loops that emerged. In this phase, which illustrates a number of events from 1995 to the present, we note many more actors and unexpected consequences – some arising with a delay – as well as the emergence of opposition to immunization.

Though opposition to the polio campaign emerged right at the start of the programme, these discussions mostly remained within academic circles. In 1999, the federal government decided to strengthen the polio eradication drive and introduced Intensified Pulse Polio Immunisation (IPPI). This included additional polio vaccination days and initiated mop-up rounds, which mandated the field workers and volunteers to reach out to unvaccinated children in their households and vaccinate them. The implementation of the programme was closely monitored by the public health authorities for providing logistics support and for achieving complete coverage.

However, some groups began seeing the IPPI programme, for which the state had mobilized significant resources, as an opportunity to bring out their own grievances. In 2002, the Kerala Government Medical Doctors’ Association (KGMOA) publically questioned the need for IPPI in Kerala and referred to the arguments raised by some medical professionals in the beginning of the program; they announced their non-cooperation with the program. The announcement coincided with a strike called by the association for better service conditions and was partly used as a bargain for their negotiations. They justified their stand in a press conference as a scientific argument. Though KGMOA had later backtracked and cooperated, this incident had triggered the first open debate on any immunization program in Kerala and emboldened many other groups, such as alternate system providers and some religious leaders, to raise objections against immunization programs (refer to reinforcing loop R1).

The public protest carried out against the IPPI campaign by the alternative medicine proponents was in fact a debate on the superiority of alternative medicine. For example, homeopathy professionals, on several occasions in the past, had direct confrontation with professionals representing allopathic medicine in Kerala. One of such conflicts started as far back as the 1970s, when allopathic providers opposed the initiation of a graduate programme in homeopathy in Kerala – described as the first of its kind anywhere in world. A leader of one of the homeopathic associations which has strong membership in northern Kerala described their campaign against IPPI programme as the payback time for the humiliation they had suffered from the hands of allopath.

“*We cannot accept it. They declare themselves that they are big people; but we don’t feel so. If all the three systems need to coexist, then there has to be mutual recognition. When we recognise allopathy as a medical system, and if they do not reciprocate it, where is the question of dialogue? That is why we not only practice* [oppose vaccinations]*, but we also preach to our patients against polio* [vaccine]*.*” *–* Homeopathic association leader Khozhikhode

The alternative medicine providers staged open protests in the north, including in the Kozhikode district. A popular health magazine of a naturopathy group had carried several articles against pulse polio immunisation, one of which was by a well-known naturopath who spoke at several public meetings against vaccines, especially the polio campaign. From 2005 onwards, opposition to IPPI from groups who opposed vaccination in general, such as homeopathy associations and experts of naturopathy, were joined by some religious organizations and was widely publicised. They often cited the opposition of IPPI by the allopathic professionals and KGMOA. This reinforced their arguments and gave credibility for their public protest (see reinforcing loop R2). Though the same groups broadcast their opposition messages across the entire state, coverage in the south was not affected by the outcry against immunization, but the northern districts began to show a decline. In the context of declining acceptability of the polio campaign, the debates that challenged the immunization programs received further credibility (refer to R3). The change in acceptability, in turn, increased public debate; especially as the negative media coverage of these events increased, thus causing a further dampening effect on vaccine acceptability (refer to balancing loop B1).

Although these frequent debates were centred on IPPI, they began to influence the community’s trust in vaccines (reinforcing loop R4). Additionally, as the incidence of vaccine-preventable diseases was drastically reduced with time, the general population felt gradual loss of fear due to relative unfamiliarity of vaccine-preventable diseases in the state. This subsequently reduced routine vaccine acceptability and created a dampening effect on vaccine acceptability (see balancing loop B2). The reinforcing loop R5 denotes a potential amplifying effect of low levels of vaccine acceptability over a long period of time on a household’s trust in vaccination. In the face of losing interest in immunization, the health workers found it difficult to convince the parents to vaccinate their children.

“*Earlier we had cases to show to people, now they are not seeing cases; it is now like a riddle to them. We now feel that the days ahead will be even tougher*” *–* Health worker (female), Kozhikode

In 2006, a death was reported after a school immunization program in Kozhikode district. As a result, there was an eruption of immediate public protest and violence against the local public health staff and facilities, as the safety of vaccines were again challenged [20, 21]. Immunisation programmes, and especially the field immunisations and school-based immunisation programmes, had to be stopped in most districts of northern Kerala.

Table 1 summarizes the critical events described above, and, in retrospect, their impact on the immunization system.

**Table 1:** **Major events and its influence on immunization coverage**

Landmark events	Period	Characteristics of the events	Impact on immunisation
EPI	1978	State supported immunisation programme, lower coverage due to lesser vaccine acceptability and supply constraints.	Low vaccination coverage.
Introduction of UIP	1985	Strong political commitment to immunisation, extensive organisation for the delivery of immunisation service, weekly immunisation.	Improvement in vaccine acceptability. Overall coverage began to improve.
Introduction of polio eradication initiative	1995	Introduced as special event and additional vaccines in addition to routine. Polio eradication drive was well received by community. Active participation of civil society in its implementation. Doubts against oral polio vaccines raised by some from public health community.	Improved coverage for routine immunisation. Successful implementation of polio eradication drive.
Last case of polio reported in Kerala	2000	Widely reported in Kerala. This was seen as a failure of public health system.	No specific impact on immunisation programme.
Opposition of IPPI immunisation strategy by KGMOA	2002	The opposition coincided with the strike call by the association. The issues were widely discussed in the media. The association backtracked after the strike was resolved.	No immediate impact on vaccination coverage, however, first open debate in Kerala on any immunisation programme.
Organised opposition against from IPPI by various groups	2005 onwards	Opposition was mainly from homeopathy groups and some experts of naturopathy, also supported by some religious organisations. Motive of continuing IPPI was openly discussed from both a technical and conspiracy angle.	Immunisation coverage in northern districts showed a decline. Coverage is intact in southern districts.
			Increased reporting of VPD cases in northern Kerala.
Death reported after school vaccination programme in a northern district	2006	Eruption of immediate public protest and violence against the local public health staff and facilities. Widespread anguish felt among public as the safety of vaccines were challenged.	Reported to have had an impact on vaccination coverage in northern districts.
Special school based Japanese encephalitis vaccination programme in Kerala	2008	Wider public debate, on the rationality and safety of vaccination programme. Media reports of vaccine side-effects impacted the programme. At the implementation level, difficulties in organising events in collaboration with schools.	Limited success in Japanese encephalitis vaccination programme.

EPI, Expanded Programme for Immunisation; IPPI, Intensified Pulse Polio Immunisation; KGMOA, Kerala Government Medical Doctors’ Association; UIP, Universal Immunization Programme; VPD, Vaccine Preventable Disease.

Though the routine immunisation programme was carried out in a similar manner, and both districts initially reached very high coverage levels, the northern district ended up with a decline in coverage for routine vaccinations. Protest against IPPI by the practitioners of alternate medicine, especially homeopathy, which is more popular in northern Kerala, was a major factor. In northern Kerala many homeopathic practitioners have actively discouraged their clients from immunising their children. Several study respondents believed that the strong influence of homeopathic medicine practitioners on households in northern Kerala helped convincing them against immunisation.

“*Because they* [homeopathy practitioner] *have a family physician status and have good relationship with some of their clients, they oppose them from accessing the mainstream* [allopathic] *system. This is the limitation of the health department personnel. We are not fully able to reach to them.*” *–* Health official (male), Kozhikode district

In northern Kerala, the popular synonym for any vaccination has always been ‘polio injection’. This pointed to the possibility that a targeted campaign against polio immunization in northern Kerala perhaps had an impact larger than its objective of opposing repeated polio drops. One of the experts interviewed reasoned that as the society did not differentiate between polio vaccines and the other vaccines, the resistance against immunization, which originally initiated against polio campaign, might have moved to routine immunization.Even though the routine vaccination was well accepted in the southern district, a school-based program for Japanese encephalitis introduced in Alappuzha district by the public health department created wide-spread public debate on the rationale and safety of vaccines. The limited success in the Japanese encephalitis program was explained by multiple media reports on increasing the side effects of the vaccine, challenges of the health department in collaborating with resistant schools, and parents refusing to send their children to school on campaign days. Though there was no immediate impact of the events on routine immunization, experts, interviewed for this study, have suggested future potential negative feedback (represented by a doted arrow in Figure 3) of such debates on routine vaccination.

### Official response to decreasing immunization coverage

An immediate state public health department’s response to the decline in the public image of immunization in northern Kerala has been to strengthen the programme which included assigning high immunization coverage targets to staff at all levels and a close scrutiny of their achievements. It was observed, for example, that during the regional review meetings, if the coverage of a particular vaccine was less than expected, a close scrutiny and justification was sought from the fieldworker and the supervisor.

Over-emphasis on coverage targets created perverse incentives for health providers to inflate their coverage estimates, and has made the immunization coverage data generated by the health department grossly unreliable as evident from several independent surveys which reported significantly lower levels of coverage. Besides, it often resulted in coercion of resistant households for vaccination. During the mop rounds for polio eradication campaign, heated exchanges between health field workers and members of households that are resistant to immunize their children were observed in many places; “*…may be because when it is forced, they may think it is for the others benefit not for their benefit*” *–* commented one of the experts who is also a district level immunization program manager.

Another strategy used in responding to the crisis was to confront with the groups that opposed immunization. For example, the public health administration, which is dominated by allopathic system of medicine, retaliated against homeopathic professionals by issuing a government order to set-up vaccine booths in government-owned homeopathy dispensaries. They described this as a strategic move and prominently projected it in a press conference and in the press statements issued on the previous day of the campaign. The homeopathy practitioners’ association found this step as intimidating and one of their office-bearers who was interviewed communicated the association’s resolve to oppose the program more strongly.The official response also included the use of community intermediaries to counter the misinformation against immunization. However, many of the field staff in Kerala had accepted that their ability to influence families in health-related decisions have reduced over the years, especially with the decline in the frequency of house visits. The introduction of community health workers known as Accredited Social Health Activists (ASHAs) has, therefore, played a positive influence in decision making of the parents on immunization. ASHA’s status as local women known to the other members of the community gave her special advantage in influencing the perceptions of community on immunization issues. During her house visits information is shared as part of a day-to-day communications. The messages related to health and immunization gets discussed and exchanged during such interactions. In addition, in their role as teacher of pre-school children, AWWs also had special access to mothers when they come to drop or pick up their children from the pre-school and used the opportunity to influence mothers’ behaviour towards immunizing their children. One of the experts interviewed had noted that the health department overlooked the potential of AWWs which resulted in gradual decline of the role of AWWs in immunization. The future potential of the right kind of interactions of AWWs and ASHAs with the households to create reinforcing loops to improve trust in vaccination is denoted by dotted arrows and loops in the Figure 3. Likewise, we also anticipate a change in disease situation due to reduction in vaccine coverage which may increase vaccine acceptability in future and improve household’s trust in vaccines.

Nevertheless, an important limitation of the strategy to use community level workers, such as ASHAs, to improve household acceptability of immunization was often observed during home visits. The mothers whose children were not vaccinated said that the decision not to vaccinate was taken by the male members of their households. “*When we speak to mothers many of them will point fingers at husband, father-in-law, or mother-in-law.*” – commented one of the paediatricians interviewed when asked about her ability to convince families who refuse vaccination. There was a greater likelihood that the male members got influenced by external factors, such as the media and the public protests, used by the alternative medicines groups. Given that the mobilization strategies used by the public health system, including ASHAs, were often designed to target mothers in the households and community, the impact of these strategies was not as intended given the important role of the male members.

## Discussion

Using a CAS lens facilitated the identification and understanding of unintended consequences and unexpected phenomena. Our CLDs illustrated the complexity underlying immunization coverage in the northern districts of Kerala and showed that the campaigns and the messages targeting against special immunization programs by some of the interest groups had consequences larger than intended as it affected households’ acceptability of routine immunization. The events that occurred at different points in time had a delayed and cumulative impact on vaccine acceptability. Our findings also showed that several informal day-to-day societal interactions within the households and community played crucial roles in creating and changing vaccine acceptability. For example, the decision making for immunization during the phase of vaccine resistance in northern Kerala showed a prominent role of male members of the households in contrast to the role played by mothers during the acceptability phase.A crucial question proposed is regarding how the public health departments governing immunization programs can retain or regain vaccine acceptability in complex contexts such as in Kerala. Our CLDs shows that several events, related or unrelated to immunization programs, affected vaccine acceptability through new actors and their interactions (Figure 3). These new interactions influenced the pathways of feedback that created vaccine acceptability in the beginning (Figure 2). The comparison of two regions in Kerala showed greater impact of feedback from actors, such as practitioners of alternate systems of medicine, negatively affecting vaccine acceptability in northern Kerala as they have stronger influence over households. Therefore, understanding what factors influenced the direction of the feedback and modulated its potential to impact the vaccine acceptability pathway assumes importance.

The study findings, as well as our review of the literature, shows ‘trust’ as an important factor that modulates this feedback between actors [22, 23]. For example, our findings reveal that from a period of suspicion and rejection, vaccines have achieved public confidence mainly through a positive feedback mechanism facilitated by its capacity to demonstrate reduction of diseases in the community (Figure 2). Information against immunization programs and the occasional reporting of vaccine-related adverse events undermined a household’s trust in immunizations. In the context of a low burden of vaccine-preventable diseases (due to several years of good vaccination coverage), the reductions in trust created a negative feedback loop that dramatically affected vaccination acceptability and coverage. We discuss our findings in the light of two theoretical interpretations of trust; trust in expert systems, and interpersonal trust, to understand the feedbacks and to explore strategies for the better governance of immunization services [22].

In the first view, acceptability to immunization in the initial phase in Kerala can be viewed as a result of the trust in institutions of professional expertise (in this case, medical knowledge) [24]. However, the conflicting messages that emerge from different systems of medicine challenge the trust which people attribute to expert systems of vaccination. The studies on vaccine resistance in other contexts, too, have noted sudden loss of trust in vaccines when counter campaigns question the scientific basis of vaccinations [25, 26]. Consistent health messages, especially from different sources of expertise, is important and can be achieved by engaging in dialogue that generates consensus rather than direct confrontation with other systems of medicine, as attempted by the public health department in Kerala. We also see a possibility of immunization programs regaining household trust when vaccine-preventable diseases reappear with the decreasing coverage (denoted by a dotted arrow with delay mark in Figure 3).

The role that the media played in such contexts in informing households with conflicting messages on immunization and how the key actors trusted each information requires attention. As observed by Giddons, plurality of information, a feature of late modernity, is a reality of many societies in the developing world and has implications on governing public health functions like immunization services [27]. For example, in Kerala, where increased penetration of 24 hours local electronic news media and several widely-read health publications, are informing households on every immunization-related side-effect and public debate on immunization programs. Sensitizing the media for more responsible reporting and using it to convey appropriate health messages are options that public health departments may use in such situations, even though it is unlikely to eliminate all unwanted information from reaching households.

In the second interpretation, trust is approached as a cognitive phenomenon or a judgment based on a rational gamble and therefore household perception of other actors’ interests is important. One of the strategies of the public health department to influence the household level decision making using community level functionaries like ASHAs, therefore, has a potential (denoted by a dotted arrow in the Figure 3). Because of the social networks in which ASHAs are interconnected, households can perceive them to serve their interests. Previous studies from other health system settings also conform this finding [28]. A study of treatment-seeking behaviour in urban Sri Lanka noted the perception of community on the motives of healthcare workers as a central factor to the formation of trust in health services, especially in the face of uncertainty about health conditions [29].

Trust in health workers can also be explained through the notions of ‘affective trust’, which is developed through emotional bonds and obligation generated through their repeated personal interactions with the households [28]. However, we observe that the trust created by community level health functionaries is intrinsically related to how they interacted with the households. In the backdrop of widespread misinformation against immunization in Kerala, when the functionaries are forced to deliver against rigid performance targets, it leads to coercive practices and may undermine the health worker’s long-term trust with the community. Thiede’s analysis of origin of trust and mistrust in healthcare draws a similar conclusion that, while trust enhanced communication of health workers, it was the process of communicative interaction that generated trust in the first place [23].

In the bureaucratic context of implementation of UIP, there was limited recognition of the need to influence informal interactions that retains trust in vaccines and in the public health department that governs immunization programs. The governance of immunization was seen as an exercise to ensure control and order through top-down hierarchical interactions. At each level, the program is conceived as an array of demands to be met. This absolves the capacity of the system to adapt to emerging complexity. Similar observations on public health bureaucracy have been made by other studies that looked at target-driven top-down implementation of public health programs in the South-East Asian context [30–32].

This study has various strengths and limitations. Using a CAS framework to guide data analysis and interpretation contributed to understanding the complexity involved in the governance of immunization services in a developing country context. It showed how system thinking concepts and methods can be applied to a complex question such as changing household acceptability to immunization. We also showed how tools such as CLDs can be used to explore social phenomena interlinked to governance of public health functions and interpret feedback loops that influenced the change in vaccination coverage. Development of CLDs based on content analysis of qualitative data and using these CLDs to guide further thinking of the complex system behaviour is arguably a unique feature of this analysis.

There are, however, several limitations to this study. Firstly, it included only two districts in the analysis of the reasons for the change in vaccination coverage in Kerala and it may not necessarily apply to other settings. Though the resistance against immunization is widespread in Kozhikode district, the intensity of resistance is varied in different regions of the district. Our description of resistance phenomenon is only pertaining to the areas that showed significant levels of resistance and cannot be applied to regions with good vaccination acceptance. This study tries to interpret and propose corrective strategies only on the basis of an ex-post analysis of complexity and therefore does not reflect on the experience of a health system that regained vaccine acceptability after losing it. For example, the study did not reflect on the changes in vaccination coverage in Alappuzha district that had occurred in the earlier phase. The CLDs could not be validated, and therefore may reflect inaccurate linkages. Additionally, it is possible that the authors omitted potentially important variables and events.

## Conclusions

We argue, through this article, that the evidence base of public health programs, such as immunizations, should go beyond epidemiological and economic analysis. Our study emphasizes the need for public health governance systems to take into consideration the nature of multiple interactions when societies organize themselves to manage a public function like immunization. This perspective goes beyond the conventional assumption that the government’s public health department is the sole governor of public health issues divorced from wider societal forces such as other key providers, social networks, and the households themselves with which the decision to vaccinate lie. It should make a careful consideration of multiple interactions involving the actors and their perceptions and ideas, which are shaped by factors such as trust.

The traditional approach to public health governance is directed by hierarchical organization designed to direct, control, and/or to even prevent interactions. However, complexity ensures that interactions will lead to unpredictable changes. The effort of the public health department should be to influence the multiple interactions of various governance actors and institutions.

## Abbreviations

AWW: Anganwadi workers
ASHA: Accredited Social Health Activist
CAS: Complex Adaptive System
CLDs: Causal loop diagrams
IPPI: Intensified Pulse Polio Immunisation
KGMOA: Kerala Government Medical Doctors’ Association
UIP: Universal Immunization Programme.
